# Impairment of eye emotion discrimination in benign childhood epilepsy with centrotemporal spikes: A neuropsychological study

**DOI:** 10.1002/brb3.2154

**Published:** 2021-05-04

**Authors:** Lulu Wu, Xinyu Yang, Kaili Zhang, Xiaocui Wang, Bin Yang

**Affiliations:** ^1^ Department of Neurology Anhui Provincial Children's Hospital Hefei China

**Keywords:** benign childhood epilepsy with centrotemporal spikes, emotion discrimination

## Abstract

**Purpose:**

To explore the characteristics of the impairment of eye emotional recognition and related clinical factors in children with benign childhood epilepsy with centrotemporal spikes (BECT).

**Methods:**

The Eye Basic Emotion Discrimination Task and Eye Complex Emotion Discrimination Task were used to study emotion discrimination in 33 recently diagnosed BECT patients and 33 BECT patients after complete remission compared to respective age‐ and gender‐matched comparison participants.

**Results:**

The scores for discrimination of sadness, fear, and disgust were significantly lower in the newly diagnosed BECT group than in the comparison group (*p* = .004, *p* = .019, and *p* = .044, respectively), while scores for recognizing happiness, anger, and surprise were not significantly different between the two groups (*p* = .248, *p* = .586, and *p* = .540, respectively). Our analysis revealed that the BECT onset age influences the scores for recognition of sadness, fear, and disgust (OR = 1.795, 95% CI: 1.097 to 2.936, *p* = .020; OR=1.846, 95% CI: 1.124 to 3.034, *p* = .016; OR = 1.851, 95% CI: 1.131–3.029, *p* = .014). After remission, the scores for discrimination of happiness, anger, sadness, fear, disgust, and surprise of the BECT group were not significantly different from the comparison group (*p* = .588, *p* = .765, *p* = .752, *p* = .984, *p* = .328, and *p* = .339, respectively).

**Conclusions:**

In our study, newly diagnosed BECT patients exhibited emotion discrimination dysfunction, mainly related to sadness, fear, and disgust, and this dysfunction was more severe the younger the age of onset was. However, after BECT remission, the ability to discriminate emotions returned to normal.

## Graphical abstract

To explore the characteristics and related factors of emotional recognition impairment in BECT patients, we conducted eye emotion discrimination studies on the newly diagnosed BECT and the postremission BECT, respectively, and found that the discrimination of sadness, fear, and disgust in newly diagnosed BECT patients has been impaired, and it is negatively correlated with the age of onset. More younger the age of onset, more severe the damage. However, there is no obvious damage to the discrimination of sadness, fear, and disgust in patients with BECT after remission. This result indicates that although BECT patients have impairment of eye emotion discrimination at the early stage of the disease, it can be returned to normal as the disease is relieved.

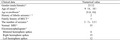

## 1 INTRODUCTION

Benign childhood epilepsy with centrotemporal spikes (BECT) is the most common epilepsy syndrome in childhood, accounting for 14%–17% of childhood epilepsy cases. The age of onset is between 3 and 13 years of age, and in almost all patients, the syndrome can be relieved before the age of 16 (Epilepsy, 1989; Guerrini & Pellacani, 2012). BECT has previously been considered a benign and self‐limiting epilepsy syndrome without associated cognitive impairment, but with the development of neuropsychology in recent years, some studies have suggested that BECT patients may experience varying degrees of cognitive impairment in areas such as attention, visual space, language expression, and decision‐making, among others cognitive functions (Besag et al., 2016; Yang et al., 2015; Vannest et al., 2013; Wickens et al., 2017). Impairment in basic cognitive functions could furthermore impair more advanced functions, such as complex social cognitive functions (Lin et al., 2012). Accurate discrimination of eye region emotions is a basic social cognitive function (Adolphs, 1999), and the ability to discriminate emotions of the eye region is a key aspect for social interaction: Accurately identifying other people's emotions can infer other people's complex inner thoughts, intentions, and attitudes, thereby guiding behavior (Adolphs, 2009). So far, it is not clear whether there is an impairment of eye region emotion discrimination during the onset period of BECT, and, importantly, whether it is resolved after remission. In this study, we therefore carried out an emotion discrimination study in BECT patients at the onset and after remission of the disease in order to explore the characteristics of potential emotional recognition impairment and its associated clinical factors in BECT patients.

## 2 METHODS

### 2.1 Participants

#### 2.1.1 Newly diagnosed children with BECT

The group of newly diagnosed patients with BECT included BECT patients diagnosed between January 2019 and December 2019 in the Department of Neurology, Anhui Provincial Children's Hospital, and participants met the following inclusion criteria: (a) conformity with the diagnostic criteria for BECT formulated by the International League Against Epilepsy (ILAE) in 1989; (b) no experience of antiepileptic treatment using drugs; (c) no history of birth injury, asphyxia, congenital diseases and related mental diseases; (d) normal mental and physical development, normal functioning of vision, hearing and color vision, and ability to understand and perform throughout the experiment. In total, 33 children with BECT, including 21 boys and 12 girls, were included in this study.

#### 2.1.2 Children with BECT after complete remission

BECT patients diagnosed in the Anhui Provincial Children's Hospital between 2009 and 2011 that met the following criteria were recruited in 2019: (a) conformity with the diagnostic criteria for BECT formulated by the International League Against Epilepsy (ILAE) in 1989; (b) the current age is over 16 years; (c) the electroencephalogram is normal; (d) no seizures for more than 2 years; (e) normal mental and physical development, normal functioning of vision, hearing and color vision, and ability to understand and perform throughout the experiment. In total, 33 children with BECT, including 22 boys and 11 girls, were enrolled.

#### 2.1.3 Comparison participants

From January 2019 to December 2019, 33 healthy participants matching the age and gender of the newly diagnosed BECT group and 33 healthy participants matching the gender and age of the remission BECT group were recruited.

This study was approved by the medical ethics committee of Anhui Provincial Children's Hospital. The guardians of all subjects fully understand the purpose and content of the study, and we obtained written informed consent from the guardians of all subjects before participation in the study.

### 2.2 Data collection

#### 2.2.1 Clinical data collection

We recorded the age of onset, age at enrollment, seizure pattern, number of seizures, electroencephalogram (EEG), and head imaging of all BECT patients at the time of their enrollment.

#### 2.2.2 Eye basic emotion discrimination task

The Eye Basic Emotion Discrimination Task (EBEDT) system (Adolphs et al., 2002) was used to study the eye emotion discrimination of 66 patients with BECT (*n* = 33 for newly diagnosed BECT patients, *n* = 33 for BECT patients with complete remission) and 66 healthy comparison participants (*n* = 33 children age‐ and gender‐matched with the newly diagnosed BECT patients, *n* = 33 children age‐ and gender‐matched with complete remission BECT patients). The test consists of 120 eye area pictures, including six basic eye area expressions: happiness, anger, sadness, fear, disgust, and surprise. The images were presented on a computer in a random manner, and each appearance included a picture of the eye area and two basic emotional words, allowing the test subject to choose the correct emotional word according to the picture. One point was counted, and the maximum score for each emotion was 20 points. The correct number of each emotion and the total number of correct emotions were calculated separately.

#### 2.2.3 Eye complex emotion discrimination task

The Eye Complex Emotion Discrimination Task (ECEDT) system (Adolphs et al., 2002) was used to study the eye emotion of 66 patients with BECT (*n* = 33 for newly diagnosed BECT patients, *n* = 33 for BECT patients with complete remission) and 66 healthy comparison participants (*n* = 33 children age‐ and gender‐matched with the newly diagnosed BECT patients, *n* = 33 children age‐ and gender‐matched with complete remission BECT patients). The test consisted of 34 black and white eye pictures (17 male, 17 female) of Chinese faces. The represented emotions included emotions such as satisfaction, worry, sadness, and expectation. The participants were asked to choose one of the four options that best described the current emotion and at the same time made a judgment on the gender of the subject in the picture. When both emotion and gender were recognized, 1 point was scored, with a potential maximum score of 34 points.

### 2.3 Statistical methods

The normality of data distributions was examined using histograms and the Shapiro–Wilk test. Data conforming to a normal distribution were expressed as mean ± *SD*. The independent sample *t* test was used for intergroup comparisons. Data that did not conform to a normal distribution were expressed as median (quartile) [*M*(Q_25_,Q_75_)], and an intergroup comparison was performed using the Mann–Whitney *U* test. Counts were expressed by the number of cases, and intergroup comparisons were carried out using chi‐square testing. Multiple ordered logistic regression was used to analyze factors associated with emotional identification impairment. All variables were initially analyzed by univariate ordered logistic regression analysis, and variables with *p* < .1 were used as covariates for multivariate ordered logistic regression. All statistics were carried out in SPSS for Windows (version 20.0). Graphs were created using GraphPad for Windows (prism 8). Statistical significance was defined as *p* < .05.

## 3 RESULTS

### 3.1 Clinical data of newly diagnosed BECT patients

The newly diagnosed BECT group consisted of a total of 33 children, including 21 males and 12 females. Clinical data are summarized in Table 1. Among the newly diagnosed BECT patients, two patients had a history of febrile seizures, and 1 had family history of BECT. All 33 patients had normal cranial imaging. The median age of onset was 9 (7–13) years, the median number of seizures was 3 (2–5), and the median age at participation in our study was 9 (7–13) years. The electroencephalogram data revealed 15 cases with right hemisphere spikes, 12 cases with left hemisphere spikes, and six cases with bilateral hemisphere spikes.

**TABLE 1:** Clinical data of newly diagnosed BECT (benign childhood epilepsy with centrotemporal spikes) patients

Clinical data	Numerical value
Gender (male/female)^a^	21/12
Age of onset^b^	9 (8, 10)
Age at enrollment^b^	[9 (8, 10)]
History of febrile seizures)^a^	2
Family history of BECT^a^	1
The number of seizures^b^	3 (3, 3.5)
Normal MRI^a^	33
Electroencephalogram^a^	
Bilateral hemisphere spikes	6
Right hemisphere spikes	15
Left hemisphere spikes	12

^a^ Count data are expressed in number of cases.

^b^ Non‐normally distributed data are expressed as [*M* (Q_25_, Q_75_)].

### 3.2 Results of the eye emotion discrimination task in the newly diagnosed BECT patient group

#### 3.2.1 Comparison of eye emotion discrimination task scores between newly diagnosed BECT patients and the comparison group

The scores for discrimination of sadness, fear, and disgust in the BECT group were significantly lower than in the age‐ and gender‐matched healthy comparison group (*p* = .004, *p* = .019, and *p* = .044, respectively), while the scores for discrimination of happiness, anger, and surprise were not significantly different between the two groups (*p* = .248, *p* = .586, and *p* = .540, respectively). There was no significant difference in the total EBEDT and ECEDT scores between the newly diagnosed BECT and comparison group (*p* = .071, *p* = .822). The results are presented in Table 2.

**TABLE 2:** The eye emotion discrimination task scores of newly diagnosed BECT patients and comparison participants ([*M* (Q25, Q75)])

Group	Happiness	Anger	Sadness	Fear	Disgust	Surprise	EBEDT	ECEDT
BECT group (*N* = 33)	19.0 (19.0, 20.0)	17.0 (14.5, 18.5)	17.0 (14.0, 17.5)	15.0 (14.0, 17.0)	17.0 (15.0, 19.0)	19.0 (16.0, 20.0)	105.0 (92.5, 107.0)	16.0 (14.5, 20.5)
Control group (*N* = 33)	19.0 (17.5, 20.0)	16.0 (15.0, 17.5)	18.0 (16.5, 18.5)	16.0 (15.0, 17.5)	18.0 (16.5, 20.0)	19.0 (16.0, 20.0)	105.0 (99.0, 110.5)	17.0 (14.0, 21.0)
*Z*	−1.156	−0.544	−2.863	−2.351	−2.011	−0.613	−1.644	−0.225
*p*	.248	.586	.004*	.019*	.044*	.540	.071	.822

* *p* ≤ .05 means the difference is statistically significant.

#### 3.2.2 Clinical factors associated with eye emotion discrimination performance in newly diagnosed BECT patients

Univariate logistic regression was used to analyze clinical factors such as gender, age of onset, number of attacks, history of febrile seizures, family history, and discharge location, which may affect eye emotion recognition in BECT patients. This revealed that gender (*p* = .039) and age of onset (*p* = .014) were associated with the sadness discrimination score, while age of onset (*p* = .014) and discharge location (*p* = .037) were associated with the fear discrimination score, and the age of onset (*p* = .014) was furthermore associated with the disgust discrimination score (Table 3).

**TABLE 3:** Results of univariate ordered logistic analysis

Variable	Sadness			Fear			Disgust		
	OR	*p*	95%CI	OR	*p*	95%CI	OR	*p*	95%CI
Gender	0.243	.039	0.065–0.931	0.537	.333	0.152–1.889	0.654	.506	0.187–2.286
Age of onset	1.844	.014	1.131–3.004	1.852	.014	1.134–3.022	1.851	.014	1.131–3.029
The number of seizures	1.592	.214	0.764–3.313	1.125	.745	0.552–2.296	1.662	.173	0.801–3.452
History of febrile seizures	0.132	.134	0.009–1.866	2.672	.446	0.214–33.348	0.164	.181	0.012–2.323
Family history of BECT	0.605	.780	0.018–20.656	0.624	.792	0.019–20.863	22.601	.114	0.473–1079.236
Discharge location	0.848	.702	0.365–1.972	0.338	.037	0.159–0.946	0.716	.437	0.308–1.664

The factors associated with eye emotion recognition discrimination in the univariate analysis were then selected as independent variables for multivariate ordered regression, while the sadness, fear, and disgust discrimination scores represented dependent variables, respectively. The results of the multivariate analysis revealed that age of onset was associated with the sadness score, with a younger onset age being associated with lower scores, and the age of onset and discharge location representing factors affecting the fear discrimination score, whereby older BECT patients with the right hemisphere spikes have relatively higher fear discrimination scores (Table 4).

**TABLE 4:** Results of multivariate ordered logistic analysis

Dependent variables	Independent variables	OR (95% CI)	*p*
Sadness	Gender	0.274 (0.072–1.047)	.058
	Age of onset	1.795 (1.097–2.936)	.020
Fear	Age of onset	1.846 (1.124–3.034)	.016
	Discharge location	0.405 (0.165–0.993)	.048

### 3.3 Clinical data of postremission BECT patients

We included 33 BECT patients after remission in our study (22 males and 11 females). The clinical data are summarized in Table 5. The median age at participation in the study was 17 (16–20) years, the median age of onset was 9 (6–12) years, the median age at the last seizure was 10 (7–13) years, and the median total seizure number was 4 (1–10). Nearly all patients (93.93%, 31 cases) were treated with antiepileptic drugs, including 18 cases treated with oxcarbazepine, three cases treated with levetiracetam, three cases treated with sodium valproate, three cases treated with oxcarbazepine and sodium valproate, two cases treated with oxcarbazepine and levetiracetam, and two cases treated with levetiracetam and sodium valproate. The median duration of antiepileptic drug treatment was 3 (2–5) years.

**TABLE 5:** Clinical data of postremission BECT patients

Clinical data	Value
Gender (male/female)^a^	22/11
Age at enrollment^b^	[17 (16, 17)]
Age of onset^b^	9.0 (8.0, 10.0)
The number of seizures^b^	4.0 (3.5, 6.0)
Age of last seizure^b^	10.0 (9.0, 11.0)
Treatment of AEDs^a^	
No anti‐epileptic drug treatment (NO)	2
OXC	18
LEV	3
VPA	3
OXC + VPA	3
OXC + LEV	2
LEV + VPA	2
Duration of antiepileptic drug therapy ^b^	3.0 (3.0,4.0)

Abbreviations: AED, antiepileptic drug; BECT, benign childhood epilepsy with centrotemporal spikes; LEV, levetiracetam; OXC, oxcarbazepine; VPA, sodium valproate.

^a^ Count data are expressed in number of cases.

^b^ Normally distributed data are expressed as [*M* (Q_25_, Q_75_)].

### 3.4 Comparison of eye emotion discrimination task scores between postremission BECT participants and the comparison group

There was no significant difference in the discrimination scores for any of the emotions in the BECT remission group compared with the healthy age‐ and gender‐matched comparison participants (happiness: *p* = .588, anger: *p* = .765, sadness: *p* = .752, fear: *p* = .984, disgust: *p* = .328, and surprise: *p* = .339). There was also no significant difference in the total EBEDT or ECEDT scores between these two groups (*p* = .680 and *p* = .887, respectively). The results are highlighted in Table 6.

**TABLE 6:** The eye emotion discrimination task scores of postremission BECT patients and comparison participants ([*M* (Q25, Q75)])

Group	Happiness	Anger	Sadness	Fear	Disgust	Surprise	EBEDT	ECEDT
Postremission BECT patients (*N* = 33)	20.0 (19.0,20.0)	19.0 (18.0,19.0)	19.0 (17.5,20.0)	17.0 (16.0,19.0)	18.0 (17.0,19.0)	20.0 (19.0,20.0)	112.0 (108.5,115.0)	24.0 (19.5,25.0)
Comparison participants (*N* = 33)	20.0 (19.5,20.0)	19.0 (18.0,19.0)	19.0 (17.0,20.0)	17.0 (15.0,19.0)	19.0 (17.0,19.0)	19.0 (18.0,20.0)	113.0 (105.0,116.0)	22.0 (21.0,25.0)
*Z*	−0.541	−0.299	−0.316	−0.019	−0.978	−0.956	−0.412	−0.142
*p*	.588	.765	.752	.984	.328	.339	.680	.887

Abbreviations: BECT, benign childhood epilepsy with centrotemporal spikes; EBEDT, Eye Basic Emotion Discrimination Task; ECEDT, Eye Complex Emotion Discrimination Task.

### 3.5 Comparison of eye emotion discrimination results between the two comparison group groups

The scores of happiness, anger, sadness, fear, EBEDT, and ECEDT in the comparison group matched with newly diagnosed BECT group were significantly lower than those of the comparison group matched with after‐remission BECT group (*p* = .001, *p* = .003, *p* = .007, *p* = .044, *p* = .001, *p* = .000), and there was no significant difference in disgust and surprise scores between the two comparison groups (*p* = .417, *p* = .158; Table 7).

**TABLE 7:** Scores of eye emotion recognition task in the two healthy comparison participant groups ([*M* (Q25, Q75)

Group	Happiness	Anger	Sadness	Fear	Disgust	Surprise	EBEDT	ECEDT
Comparison participants matched with newly diagnosed BECT								17.0 (14.0,21.0)
Comparison participants matched with postremission BECT (*N* = 33)	20.0 (19.5,20.0)	19.0 (18.0,19.0)	19.0 (17.0,20.0)	17.0 (15.0,19.0)	19.0 (17.0,19.0)	19.0 (18.0,20.0)	113.0 (105.0,116.0)	22.0 (21.0,25.0)
*Z*	−3.181	−2.988	−2.674	−2.016	−0.811	−1.410	−3.473	−4.480
*p*	.001	.007	.007	.044	.417	.158	.001	.000

Abbreviations: BECT, benign childhood epilepsy with centrotemporal spikes; EBEDT, Eye Basic Emotion Discrimination Task; ECEDT, Eye Complex Emotion Discrimination Task.

## 4 DISCUSSION

BECT is a common childhood epilepsy syndrome. Previous studies have suggested that patients with BECT may exhibit an impairment of recognition of fear and disgust (Ciumas et al., 2017), but no comprehensive study of emotion recognition has been conducted in BECT patients after remission. Our study was therefore the first to explore and contrast emotion recognition in newly diagnosed BECT patients and BECT patients after remission.

In contrast to previous studies which showed pictures of faces to participants, we only showed eye region pictures to participants in our study as eye emotion recognition requires higher cognitive understanding than facial emotion recognition, and it also requires regulation of the top‐down cognitive processing functions. Our results showed that there was no significant difference in the recognition of happiness, anger, and surprise between the newly diagnosed BECT patients and the comparison group, while the recognition of sadness, fear, and disgust was significantly lower in newly diagnosed BECT patients than in the comparison group. Previous studies found that the most common cognitive impairment in focal epilepsy was a recognition defect of fear emotions, followed by recognition defects of sadness and disgust (Monti & Meletti, 2015), while recognition of anger and surprise was less impaired, and there was essentially no impairment in the recognition of happiness. These previous findings are consistent with the results of our current study. The insular cortex is involved in processing fear and aversion, and the dorsal striatum plays an important role in the perception of fear (Paulus & Stein, 2006; Samejima et al., 2005). Morphological studies showed that the thickening of the insular cortex and striatal hypertrophy are delayed in BECT patients (Kim et al., 2015; Pardoe et al., 2013), which may be related to the observed impairment in the recognition of sadness, fear, and disgust in newly diagnosed BECT patients.

Our study also revealed a significant positive correlation between the age of onset and the eye emotion discrimination scores, with a younger age of onset corresponding to lower recognition score of sadness, fear, and disgust: the younger the onset age, the more severe the impairment. Previous studies have found that early seizures may affect the recognition of various negative emotions, and the younger the age, the more severe the impairment of negative emotion recognition (Monti & Meletti, 2015), which may be related to the delayed development of neural networks involved in social cognition (Ciumas et al., 2017). Fuerst et al., (2003) identified a negative correlation between the sadness emotion recognition score and the number of seizures. Patients experiencing more seizures exhibit more severe damage to the microstructure of the brain and synaptic connections between adjacent neurons, which may result in more frequent seizures and a more serious emotion recognition damage of sadness. However, in our study, we found no significant association between the number of seizures and eye emotion recognition scores. Baron‐Cohen et al. found that women may have superior facial expression recognition (Baron‐Cohen & Wheelwright, 2004), and the authors found that sadness recognition score is related to gender, with the scores of females being higher in males. Previous studies have found that patients with right brain injury have severe emotional recognition impairments. However, it has also been found that patients with left brain injury have severe emotional recognition impairments, which means that both the left and right hemispheres may be involved in emotion recognition, but there is no unified conclusion yet on which one is more important (Monti & Meletti, 2015). Our study found that fear recognition is indeed related to the location of spikes and that emotion recognition impairment is lower with spikes in the right hemisphere than in the left hemisphere.

In this study, we also conducted eye emotion discrimination tests in BECT patients after remission and found that these patients exhibited no significant differences in eye emotion discrimination for emotions such as happiness, anger, sadness, fear, disgust, surprise, and more eye complex emotion discrimination compared to the age‐matched comparison group. This is in line with previous findings by Völkl‐Kernstock et al. (2009), who reported that cognitive problems were reversible in most children after remission of seizure activity. Moreover, Camfield and Camfield (2017) found that some academic or behavioral problems of BECT patients are resolved during later stages of the disease. Conducting fMRI in BECT patients at onset, during adolescence and in adulthood, Pardoe et al. (2013) found an increased thickness and gray matter volume in the frontal, insular lobe, and parietal lobe of BECT patients at onset of the disease, but these abnormalities disappeared after BECT remission and former BECT patients exhibited no significant differences from the healthy controls with respect to cortical thickness and gray matter volume in adulthood. This may help to explain the fact that neuropsychological impairments present during early stages of BECT are resolved after BECT remission.

In this study, the eye emotion discrimination scores of the two comparison groups were also compared. The median age of the participants in the comparison group matched with the newly diagnosed BECT group was 9 (8–10) years, while the median age of the participants in the comparison group matched with the postremission BECT group was 17 (16–17). We found that the comparison group matched with the newly diagnosed BECT group had significantly lower eye emotion discrimination scores than the comparison group matched with the postremission BECT group. The research results also showed that the emotion discrimination ability of teenagers is generally significantly better than that of school‐age children; this is in line with previous findings which suggested that emotion discrimination in children improves with age (Chronaki et al., 2015). Facial emotion processing continues to develop from the preschool years through to middle childhood and adolescence (Herba et al., 2006). Similarly, research has shown that the neural substrates involved in facial emotion processing are not adult‐like until early adolescence (Batty & Taylor, 2006). Compared with adults, children may not be able to identify happiness, fear, and sadness as accurately (Chronaki et al., 2013; Gao & Maurer, 2009; Montirosso et al., 2010). In this study, we also found no significant difference in the ECEDT scores in newly diagnosed BECT patients compared with healthy comparison participants. This may be due to the relatively young age of the newly diagnosed BECT and the comparison group, at which the eye complex emotion discrimination system is not fully mature, and neither group can accurately discriminate complex emotions.

There were some limitations to our current study. First, this study is limited primarily by its relatively small sample size and mismatching of ages between the patient groups and the inability to compare the two patient groups directly, which may hinder its generalization. Second, this study mainly focused on the emotion recognition of newly diagnosed BECT patients and BECT patients after remission, but did not include BECT patients during treatment process. Although we have studied the effects of the age of onset, the number of seizures, and brain discharge on emotion recognition, we have not studied the effect of antiepileptic drugs on emotion recognition. Future studies will include BECT patients in the process of treatment to study the effects of antiepileptic drug treatment on emotion recognition. Third, we did not conduct a detailed study on typical BECT and variant BECT. Variant BECT patients exhibit a more serious cognitive impairment which may affect emotion recognition. Besides, this current study was based entirely on behavior, but the mechanisms underlying emotion recognition impairment may be explored by combining neuroimaging and electrophysiological technologies in the future.

## 5 CONCLUSIONS

In our study, newly diagnosed BECT patients had emotions discrimination dysfunction, mainly relating to the emotions sadness, fear, and disgust. This dysfunction was more severe the younger the age of onset was. However, after BECT remission, the emotions discrimination dysfunction of patients returned to normal.

## CONFLICTS OF INTEREST

None declared.

### PEER REVIEW

The peer review history for this article is available at https://publons.com/publon/10.1002/brb3.2154.
